# Reliability of the Amharic Version of the Medication Adherence Report Scale and Beliefs about Medicines Questionnaire in Patients with Asthma in Ethiopia

**DOI:** 10.4314/ejhs.v35i1.3S

**Published:** 2025-12

**Authors:** Girma Tekle Gebremariam, Markos Hamiso Hayiso, Zoe Moon, Rob Horne, Hanan Yusuf, Rahel Argaw Kebede, Eskinder Eshetu Ali, Bruck Messele Habte

**Affiliations:** 1 School of Pharmacy, College of Health Sciences, Addis Ababa University, Addis Ababa, Ethiopia; 2 Centre for Behavioural Medicine, School of Pharmacy, University College London, London, UK; 3 School of Medicine, College of Health Sciences, Addis Ababa University, Addis Ababa, Ethiopia

**Keywords:** Asthma, Adherence to medication, Ethiopia, Reliability, MARS-A, BMQ-Specific

## Abstract

**Background:**

Non-adherence to asthma medications is often associated with poor health outcomes, necessitating a reliable measurement of its extent. Thus, the aim of this study is to assess the reliability of the Medication Adherence Report Scale for asthma (MARS-A) and the Beliefs about Medicines Questionnaire–Specific (BMQ-Specific) adherence measures among patients with asthma in Ethiopia.

**Methods:**

A hospital-based cross-sectional study was conducted among adult patients with asthma at a tertiary hospital in Ethiopia from January to May 2024. The MARS-A and BMQ-Specific tools were used to assess adherence and patients' beliefs about medicines, respectively, while sociodemographic and clinical characteristics, as well as reasons for non-adherence, were obtained through patient interviews and electronic medical records. Descriptive statistics were used to present patient characteristics, and Cronbach's alpha was calculated to evaluate internal consistency.

**Results:**

The study included 250 patients with a mean age of 53.82 (SD = 13.79) years, of whom 57.2% had well-controlled asthma, and 24.8% were non-adherent to prescribed medications according to MARS-A. According to patient reports, the primary reasons for non-adherence were unaffordability and unavailability of medications. The overall mean (SD) MARS-A score was 4.60 (0.43). The MARS-A and BMQ-Specific instruments were found to be reliable, with Cronbach's alphas of 0.904 and 0.961, respectively.

**Conclusion:**

Our findings indicate that the Amharic versions of the MARS-A and BMQ-Specific are reliable instruments for assessing medication adherence and beliefs about medicines among Ethiopian patients with asthma.

## Introduction

More than 300 million people are affected by asthma worldwide, with the number expected to surpass 400 million by 2025 (1). In Ethiopia, about 8 percent of the population is affected by asthma, which is associated with considerable morbidity, mortality, and economic burden for patients and the healthcare system (2). Adherence to prescribed controller asthma medications is crucial for controlling symptoms and preventing frequent attacks. However, adherence rates remain suboptimal (3–8), with studies reporting adherence ranging from 30% to 70% (9–11), which subsequently contributes to uncontrolled asthma (10,12–14). Similar adherence levels have been reported in studies from Ethiopia (6,10). Patient age; lack of awareness about the disease; beliefs about medicines; regimen complexity; high medication costs; comorbidities; adverse effects; and poor communication with healthcare providers have all been cited as factors influencing patients' adherence to asthma medications (15–18). Non-adherence often leads to uncontrolled symptoms (8).

Patient beliefs and attitudes toward their medications play an important role in adherence behavior (19–21). Therefore, healthcare providers should engage patients in shared decision-making, as these factors strongly influence adherence (22). This can be achieved through reliable evaluation of patients' beliefs, attitudes, and adherence behavior. In this regard, the Beliefs about Medicine Questionnaire-Specific (BMQ-Specific) is a widely used tool for assessing patients' beliefs about their prescribed medications (23). It considers personal views on medication necessity and concerns about potential adverse effects (21). Additionally, several self-reported measurement tools have been developed to assess patient adherence in routine clinical practice and research (24–27). The Medication Adherence Report Scale for Asthma (MARS-A) is one of the most widely used self-reporting tools and has been validated in various countries to measure asthma medication adherence, although its reliability has not been established in Ethiopia (24,28). Hence, our study aimed to assess the reliability of the two instruments among patients with asthma in Ethiopia. The results will provide reliable and culturally adapted tools for clinicians and researchers to evaluate adherence and patient beliefs about medications, thereby helping address gaps in adherence.

## Methods

Study design and setting: This was a hospital-based cross-sectional study conducted from January to May 2024 among patients with asthma attending the outpatient chest clinic of Tikur Anbessa Specialized Hospital, a tertiary care teaching hospital in Addis Ababa with over 800 beds and serving more than half a million patients annually.

**Participant recruitment and sampling**: Patients visiting the chest clinic who met the eligibility criteria were approached consecutively. Eligibility criteria included being on regular follow-up for at least 6 months, using inhaled corticosteroids, being older than 18 years, and providing written informed consent. Patients who declined to participate or were acutely ill to be interviewed were excluded. For this study, the 11-item BMQ-Specific questionnaire was used to determine a minimum sample size of 220 patients with asthma. To account for potential non-response, a 12% contingency was added, resulting in a final sample size of 250 patients.

**Data collection procedures**: Two trained clinical pharmacists conducted face-to-face interviews using interviewer-administered tools. Written informed consent was obtained from all participants. Sociodemographic information—such as sex, age, marital status, occupation, education level, alcohol consumption, smoking status, and precipitating factors—was collected. Clinical characteristics (duration of asthma, comorbidities, and the number of medications taken) were obtained from electronic medical records, while reasons for non-adherence (including unaffordability, unavailability, forgetfulness, and difficulty using inhalers) were obtained through interviews. The MARS-A and BMQ-Specific were used to assess adherence and beliefs about medicines, respectively.

**MARS-A and BMQ-Specific Instruments**: The MARS-A is a 10-item questionnaire with strong psychometric properties (24). It assesses both intentional and unintentional non-adherence. For example, “I try to avoid using it” measures intentional non-adherence, while “I forget to take it” evaluates unintentional non-adherence. The tool uses negatively framed statements to reduce social desirability bias. Patients rate how often they engage in each non-adherent behavior using a 5-point Likert scale (5 = Never, 4 = Rarely, 3 = Sometimes, 2 = Often, 1 = Always). Adherence is calculated as the average score of all 10 items, ranging from 0 to 5. A score ≥ 4.5 indicates good adherence (24,29).

The BMQ-Specific is an 11-item questionnaire consisting of two domains: Necessity (“Specific-Necessity”) and Concerns (“Specific-Concern”). The Necessity scale measures perceived need for medication, while the Concerns scale addresses worries about potential adverse outcomes. Each item is scored on a five-point Likert scale (1 = strongly disagree to 5 = strongly agree). Higher scores reflect stronger perceptions of the respective domain (23).

**Translation**: Translation into Amharic was conducted after obtaining permission from the measure's originator. Two bilingual professionals translated both tools, and two others conducted a back-translation following the originator's recommendations. The original English versions were translated independently, combined into a single version, and then back-translated. The final English and Amharic versions were sent to the originator for comments and authorization.

**Statistical analyses**: Descriptive statistics were used to present patient characteristics and MARS-A and BMQ-Specific data. A chi-square test assessed associations between patient characteristics and adherence status measured by MARS-A. Cronbach's alpha and Cronbach's alpha if item deleted were calculated to evaluate internal consistency, with values > 0.70 considered satisfactory (30). Cronbach's alpha values of 0.6–0.7 indicated acceptable reliability; 0.7–0.8 satisfactory reliability; and ≥ 0.8 excellent reliability. Construct validity was assessed by examining correlations between MARS-A and asthma control as well as BMQ-Specific domains. Analyses were conducted using R version 4.4.1, with significance set at p < 0.05.

## Results

**Sociodemographic and clinical characteristics**: A total of 250 patients participated, with a mean age of 53.82 years (SD = 13.79). The majority were female (167, 66.8%), and 26 (10.4%) were illiterate. Older adults constituted over one-third of the sample (99, 39.6%), and most participants (182, 72.8%) were employed. The median monthly household income was $104.51 (based on an exchange rate of $1 = 57.41 ETB), and most participants (184, 73.6%) used Community-Based Health Insurance (CBHI) (Table 1).

**Table 1:** Sociodemographic characteristics of the participants (N= 250)

Variables	All patients N (%)	Adherent N (%)	Non-adherent N (%)	p-value
Age, mean (SD)	53.82 (13.79)			
Gender				
Male	83 (33.2)	59 (31.4)	24 (38.7)	0.288
Female	167 (66.8)	129 (68.6)	38 (61.3)	
Age category				
18-39	44 (17.6)	35 (18.7)	8 (12.9)	0.044*
40-59	107 (42.8)	86 (46.0)	21 (33.9)	
> 60	99 (39.6)	66 (35.3)	33 (53.2)	
Marital status				
Married	202 (80.8)	157 (83.5)	45 (72.6)	0.129
Single	16 (6.4)	11 (5.9)	5 (8.10	
Divorced	25 (10.0)	17 (9.0)	8 (12.9)	
Widowed	7 (2.8)	3 (1.6)	4 (6.5)	
Educational status				
Illiterate	26 (10.4)	17 (9.0)	9 (14.5)	0.002*
Able to read and write	23 (9.2)	12 (6.4)	11 (17.7)	
Primary school	33 (13.2)	22 (11.7)	11 (17.7)	
Secondary school	74 (29.6)	55 (29.3)	19 (30.6)	
Higher education	94 (37.6)	82 (43.6)	12 (19.4)	
Employment status				
Employed	182 (72.8)	144 (76.6)	38 (61.3)	0.026*
Non-employed	68 (27.2)	44 (23.5)	24 (38.7)	
Place of residence				
Addis Ababa	226 (90.4)	173 (92.0)	53 (85.5)	0.13
Outside of Addis Ababa	24 (9.6)	15 (8.0)	9 (14.5)	
Monthly household income				
1st quartile	0-4000			
2nd quartile	4001-6000			
3rd quartile	6001-9000			
Payment method				
CBHI	184 (73.6)	133 (70.7)	51 (82.3)	0.16
Non-CBHI users	66 (26.4)	55 (29.3)	11 (17.7)	
Smoking status				
No	246 (98.4)	187 (99.5)	59 (95.2)	0.019*
Yes	4 (1.6)	1 (0.5)	3 (4.8)	
Alcohol habit				
No	189 (75.6)	152 (80.9)	37 (59.7)	0.001*
Yes	61 (24.4)	36 (19.1)	25 (40.3)	
Khat				
No	246 (98.6)	185 (98.4)	61 (98.4)	0.993
Yes	4 (1.6)	3 (1.6)	1 (1.6)	
Family history of asthma				
No	238 (95.2)	182 (96.8)	56 (90.3)	0.038*
Yes	12 (4.8)	6 (3.2)	6 (9.7)	

SD: Standard deviation; COPD: Chronic Obstructive Pulmonary disease; CBHI: Community Based health insurance

Most patients (151, 60.4%) had been living with asthma for more than 10 years. In terms of severity, 162 (64.8%) had mild intermittent asthma, and 45 (18.0%) had mild persistent asthma. A large proportion (203, 81.2%) had comorbid conditions, averaging 2.36 per patient, with lung-related comorbidities accounting for 41 (20.2%). The most common regimen was salbutamol as needed plus beclomethasone twice daily (100, 40.0%), followed closely by budesonide with formoterol plus salbutamol (98, 39.2%). Asthma was well controlled in 143 (57.2%) patients. Reasons for non-adherence included medication unaffordability and unavailability. Additionally, 88 (35.2%) experienced exacerbations in the past year, triggered mainly by environmental pollution, infections, physical activity, and stress (Table 2).

**Table 2:** Clinical characteristics of the participants (N=250)

Variables	All patients N (%)	Adherent N (%)	Non-adherent N (%)	p-value
Duration of asthma, year				
<5	66 (26.4)	47 (25.0)	19 (30.6)	0.649
5-10	33 (13.2)	26 (13.8)	7 (11.3)	
> 10	151 (60.4)	115 (61.2)	36 (58.1)	
Severity of asthma				
Mild intermittent	162 (64.8)	131 (69.7)	31 (50.0)	0.001*
Mild persistent	45 (18.0)	38 (20.2)	7 (11.3)	
Moderate persistent	24 (9.6)	11 (5.9)	13 (21.0)	
Severe persistent	19 (7.6)	8 (4.3)	11 (17.7)	
Presence of comorbidities				
No	47 (18.8)	36 (19.1)	11 (17.7)	0.806
Yes	203 (81.2)	152 (80.9)	51 (82.3)	
Number of comorbidities, mean (SD)	2.36 (1.42)			
Lung related comorbidities				
No	162 (79.8)	121 (79.6)	38 (74.5)	0.445
Yes	41 (20.2)	31 (20.4)	13 (25.5)	
Types of lung related comorbidities				
Tuberculosis and its sequalae	20 (48.78)	14 (34.15)	6 (14.63)	0.382
COPD	10 (24.39)	5 (12.20)	5 (12.20)	
hypersensitivity pneumonitis	4 (9.78)	3 (9.7)	1 (2.44)	
Tuberculosis and its sequalae + COPD	7 (17.1)	6 (17.02)	1 (2.44)	
Current treatment				
Salbutamol puff + Beclomethasone puff	100 (40.0)	78 (41.5)	22 (35.5)	0.002*
Budesonide + Formoterol + Salbutamol puff	98 (39.2)	78 (41.5)	20 (32.3)	
Budesonide + Formoterol	22 (8.8)	17 (9.0)	5 (8.1)	
Salbutamol puff alone	9 (3.6)	1 (0.5)	8 (12.9)	
Salbutamol puff + Beclomethasone puff + Prednisolone	6 (2.4)	4 (2.1)	2 (3.2)	
Budesonide + Formoterol + Salbutamol puff + Prednisolone	5 (2.0)	3 (1.6)	2 (3.2)	
Budesonide + Formoterol + Beclomethasone puff	5 (2.0)	4 (2.1)	1 (1.6)	
Others	5 (2.0)	3 (1.5)	2 (3.2)	
Treatment outcome				
Well controlled	143 (57.2)	127 (67.6)	16 (25.8)	0.001*
Partially controlled	85 (34.0)	56 (29.8)	29 (46.8)	
Uncontrolled	22 (8.8)	5 (2.7)	17 (27.4)	
Reason for non-adherence				
Unaffordability of medications	9 (40.9)	0 (0.0)	9 (52.9)	0.013*
Unavailability of medications	4 (18.2)	1 (20.0)	3 (17.6)	
Unaffordability + unavailability of medications	3 (13.6)	0 (0.0)	3 (17.6)	
Unavailability of medications + difficulty using inhalers	2 (9.1)	0 (0.0)	2 (11.8)	
Unknown	4 (18)	4 (80.0)	0 (0.0)	
Presence of exacerbation in the past 12 months				
No	162 (64.8)	134 (71.3)	28 (45.2)	0.001*
Yes	88 (35.2)	54 (28.7)	34 (54.8)	
Drug discontinuation in the past 12 months				
No	216 (86.4)	179 (95.2)	37 (59.7)	0.001*
Yes	34 (13.6)	9 (4.8)	25 (40.3)	
Hospital admission in the past 12 months				
No	218 (87.2)	177 (94.1)	41 (66.1)	0.001*
Yes	32 (12.8)	11 (5.9)	21 (33.9)	
Precipitating factors				
Seasonal variation	29 (11.6)	26 (13.8)	3 (4.8)	0.018*
Seasonal variation + infections	36 (14.4)	24 (12.8)	12 (19.4)	
Seasonal variation + infections + pollution	8 (3.2)	5 (2.7)	3 (4.8)	
Infections + pollution	12 (4.8)	10 (5.3)	2 (3.2)	
Seasonal variation + pollution	13 (5.2)	9 (4.8)	4 (6.5)	
Infections + pollution + exercise	85 (34.0)	58 (31.8)	27 (43.5)	
Pollution + exercise + stress	67 (26.8)	56 (29.8)	11 (17.7)	

**Description of MARS-A**: The overall mean (SD) adherence score was 4.60 (0.43). A total of 188 (75.2%) patients had high adherence (≥ 4.5). Most patients reported “never” engaging in non-adherent behaviors, such as using medication only when feeling breathless (88.8%). Only small proportions reported non-adherent behaviors such as using medication as a backup when other treatments failed (1.6%) or adjusting the dose (2.8%) (Table 3).

**Table 3:** Participants mean score, percentage of each item, and overall adherence (N= 250)

Items How often do you…	Mean (SD)	Number (%)				

		Always	Often	Sometimes	Rarely	Never
I only use my medicine when I need it	4.82 (0.61)		9 (3.6)	1 (0.4)	17 (6.8)	223 (89.2)
I only use it when I feel breathless	4.80 (0.65)		11 (4.4)		17 (6.8)	222 (88.2)
I decided to miss out a dose	4.82 (0.51)		1 (0.4)	11 (4.4)	1 (8.0)	218 (87.2)
I try to avoid using it	4.82 (0.51)		2 (0.8)	8 (3.2)	22 (8.8)	218 (87.2)
I forget to take it	4.05 (0.69)		5 (2.0)	38 (15.2)	147 (58.8)	60 (24)
I alter the dose	4.81 (0.46)			7 (2.8)	33 (13.2)	210 (84.0)
I stop taking it for a while	4.77 (0.57)		4 (1.6)	6 (2.4)	33 (13.2)	207 (82.8)
I use it as a reserve, if my other treatment doesn't work	4.88 (0.38)			4 (1.6)	23 (9.2)	223 (89.2)
I use it before doing something which might make me breathless	3.38 (0.85)	4 (1.6)	40 (16.0)	72 (28.8)	125 (50.0)	9 (3.6)
I take it less than instructed	4.82 (0.47)		1 (0.4)	6 (2.4)	31 (12.4)	212 (84.8)
Overall mean (SD) score of MARS-A	4.60 (0.43)					
Overall adherence	High adherence: 188 (75.2%)					
	Low Adherence: 62 (24.8 %)					

**BMQ-Specific**: Mean (SD) scores for Necessity and Concern were 4.50 (0.63) and 4.38 (0.66), respectively. A majority (177, 70.8%) strongly agreed that their current health depends on their asthma medicines, and 162 (64.8%) strongly agreed that life is not possible without them. Similarly, 165 (66.0%) strongly agreed that they would get very sick without their medications. Additionally, 151 (60.4%) strongly agreed that they worry about long-term effects and becoming dependent on their medicines (Table 4).

**Table 4:** Overall mean score of BMQ-Specific, necessity-specific, concern-specific, and percentage of each item (N= 250)

BMQ-Specific items	Mean(SD)	Number (%)				

		Strongly Agree	Agree	Uncertain	Disagree	Strongly Disagree
My health at present depends on my asthma medicines	4.68(0.54)	177 (70.8)	68(27.2)	3 (1.2)	2 (0.8)	
I am worried about taking my asthma medicine	4.56(0.60)	151 (60.4)	93 (37.2)	2 (0.8)	4 (1.6)	
Life is not possible without my asthma medicine	4.59(0.64)	162 (64.8)	80 (32.0)	2 (0.8)	6 (2.4)	
Sometimes I worry about the long-term effects	4.58(0.54)	151 (60.4)	93 (37.2)	6 (2.4)	0	
I get very sick without my asthma medicine	4.57(0.67)	165 (66.0)	67 (26.8)	13 (5.2)	5 (2.0)	
My asthma medicine is a mystery for me	4.39(0.74)	128 (51.2)	98 (39.2)	17 (6.8)	7 (2.8)	
My future health is dependent on my asthma medicine	4.10(1.06)	123 (49.2)	60 (24.0)	36 (14.4)	31 (12.4)	
Asthma medicine is disrupting my life	4.18(0.99)	117 (46.8)	90 (36.0)	13 (5.2)	30 (12.0)	
Sometimes I worry about becoming overly dependent on medicine	4.41(0.65)	122 (48.8)	110 (44.0)	16 (6.4)	2 (0.8)	
My asthma medicine keeps my illness from getting worse	4.56(0.63)	156 (62.4)	80 (32.0)	12 (4.8)	2 (0.8)	
My asthma medicine is causing me unpleasant side effects	4.18(0.91)	116 (46.4)	79 (31.6)	40 (16.0)	15 (6.0)	
Overall mean (SD) score of BMQ-Specific	4.40(0.63)					
Overall mean (SD) score of necessity-specific	4.50(0.63)					
Overall mean (SD) score of concern-specific	4.38(0.66)					

**Reliability of MARS-A and BMQ-Specific**: The Cronbach's alpha coefficient for MARS-A was 0.904. Coefficients if items were deleted ranged from 0.88 to 0.92. Inter-item correlations were high; for example, “I use my asthma medicine only when I need it” correlated strongly (0.95) with “I only use it when I feel breathless,” indicating that these items measure the same construct. However, “I forget to take it” had relatively lower correlations with most other items (0.26–0.40). Deleting certain items—such as “I use it before doing something which might make me breathless”—would slightly increase the overall alpha (Table 5). Nonetheless, the full scale demonstrated excellent reliability.

**Table 5:** Inter-item correlation, overall Cronbach's Alpha, and Cronbach's Alpha if Item Deleted of MARS-A and BMQ-Specific

Item/variable	I only use my medicine	I only use it when I feel breathless	I decide to miss out a dose	I try to avoid using it	I forget to take it	I alter the dose	I stop taking it for a while	I use it as a reserve, if my other treatment doesn't work	I use it before doing something which might make me breathless	I take it less than instructted	Cronbach's Alpha if Item Deleted
I only use my medicine	1.000										0.884
I only use it when I feel breathless	0.95	1.000									0.884
I decided to miss out a dose	0.74	0.78	1.000								0.884
I try to avoid using it	0.71	0.75	0.95	1.000							0.885
I forget to take it	0.27	0.26	0.28	0.29	1.000						0.914
I alter the dose	0.42	0.39	0.47	0.46	0.35	1.000					0.899
I stop taking it for a while	0.70	0.70	0.84	0.85	0.37	0.55	1.000				0.882
Use it as a reserve, if my other treatment doesn't work	0.77	0.78	0.83	0.77	0.26	0.47	0.75	1.000			0.892
I use it before doing something which might make me breathless	0.39	0.38	0.35	0.31	0.28	0.26	0.36	0.29	1.000		0.922
Take it less than instructed	0.58	0.56	0.63	0.65	0.40	0.79	0.73	0.60	0.27	1.000	0.891
Overall Cronbach's Alpha											0.904

Item	BS1	BS2	BS3	BS4	BS5	BS6	BS7	BS8	BS9	BS10	BS11	Cronbach's Alpha if Item Deleted
BS1	1.000											0.959
BS2	0.67	1.000										0.959
BS3	0.87	0.73	1.000									0.957
BS4	0.68	0.87	0.75	1.000								0.959
BS5	0.79	0.57	0.82	0.61	1.000							0.958
BS6	0.68	0.82	0.75	0.80	0.68	1.000						0.955
BS7	0.64	0.64	0.69	0.61	0.68	0.78	1.000					0.957
BS8	0.59	0.67	0.66	0.64	0.65	0.80	0.89	1.000				0.957
BS9	0.69	0.69	0.75	0.72	0.76	0.80	0.84	0.84	1.000			0.955
BS10	0.72	0.51	0.73	0.53	0.82	0.68	0.78	0.76	0.80	1.000		0.957
BS11	0.63	0.65	0.69	0.63	0.70	0.75	0.90	0.87	0.86	0.77	1.000	0.955
Overall Cronbach's Alpha												0.961

BS: refer the 11 items; BMQ: Beliefs about Medicines Questionnaire

Cronbach's alpha for BMQ Necessity and Concern scales was 0.92 and 0.94, respectively. Inter-item correlations were also high. for example, “Life is not possible without asthma medicine” correlated strongly (0.87) with “Health at present depends on asthma medicines,” while “Future health depends on asthma medicine” correlated strongly (0.89) with “Asthma medicine is disrupting life” (Table 5).

**Construct validity**: Construct validity was assessed by examining correlations between adherence and beliefs. The BMQ-Specific Necessity score was significantly positively correlated with MARS-A adherence (r = 0.33, p = 0.001), while the BMQ-Concerns score was significantly negatively correlated with adherence (r = -0.391, p = 0.001). A significant inverse correlation was also found between adherence and asthma control (r = -0.482, p = 0.001), indicating that lower adherence was associated with poorer control.

## Discussion

This study established that the Amharic versions of MARS-A and BMQ-Specific are reliable for measuring self-reported adherence and beliefs about medicines in Ethiopia. The Cronbach's alpha for MARS-A (0.904) aligns with findings from studies using the MARS-5 version (24,31). The BMQ-Specific also showed strong reliability, with alpha values of 0.916 (Necessity) and 0.940 (Concerns), consistent with findings on the Arabic translation (31). These values are higher than those reported in some other studies (32), supporting the contextual reliability of both tools for Amharic-speaking patients.

The high mean adherence score (4.60 [0.43]) suggests generally good adherence. About 75.2% of participants demonstrated good adherence, higher than in the study by Liu et al. (33). This may reflect cultural tendencies to overreport adherence due to concerns about disappointing healthcare providers or fear of judgment. Nonetheless, some non-adherent behaviors were noted, such as forgetting doses.

Findings from the BMQ indicate a strong perceived necessity for asthma medicines; however, patients also reported substantial concerns about long-term effects and dependence, which may hinder adherence. This underscores the need for patient-centered communication and shared decision-making.

Consistent with other studies (19,34), higher perceived necessity was associated with higher adherence, while greater concerns were associated with lower adherence. These findings align with established theoretical frameworks linking beliefs and adherence (28,35,36). Clinically, this suggests that healthcare professionals should proactively explore and address patients' concerns to foster better adherence.

This is the first study to assess the reliability of both MARS-A and BMQ-Specific in a large sample of Amharic-speaking patients with asthma. However, limitations include lack of test–retest reliability assessment and the inherent risk of social desirability bias in self-report tools.

Overall, the findings demonstrate that the Amharic versions of MARS-A and BMQ-Specific are reliable instruments for measuring medication adherence and beliefs about medicines among patients with asthma. Further psychometric evaluation across diverse cultural groups is recommended.

## References

[1] (2022). The Global Asthma Report 2022. Int J Tuberc Lung Dis.

[2] Mulugeta T, Ayele T, Zeleke G, Tesfay G (2022). Asthma control and its predictors in Ethiopia: Systematic review and meta-analysis. PLoS One.

[3] Amin S, Soliman M, McIvor A, Cave A, Cabrera C (2020). Understanding patient perspectives on medication adherence in asthma: A targeted review of qualitative studies. Patient Prefer Adherence.

[4] Venkatesan P (2023). 2023 GINA report for asthma. Lancet Respir Med.

[5] Asamoah-Boaheng M, Bonsu KO, Farrell J, Oyet A, Midodzi WK (2021). Measuring medication adherence in a population-based asthma administrative pharmacy database: A systematic review and meta-analysis. Clin Epidemiol.

[6] Zewdie S, Mekuria B, Alemu BK, Bayked EM, NurAhmed Toleha H, Ayenew W (2024). Prevalence of medication adherence among adult asthmatic patients in four African countries: A systematic review and meta-analysis. World Allergy Organ J.

[7] Bidwal M, Lor K, Yu J, Ip E (2017). Evaluation of asthma medication adherence rates and strategies to improve adherence in the underserved population at a Federally Qualified Health Center. Res Soc Adm Pharm.

[8] Aberhe W, Hailay A, Zereabruk K, Mebrahtom G, Haile T (2020). Non-adherence to inhaled medications among adult asthmatic patients in Ethiopia: a systematic review and meta-analysis. Asthma Res Pract.

[9] Sofianou A, Martynenko M, Wolf MS, Wisnivesky JP, Krauskopf K, Wilson EAH (2013). Asthma beliefs are associated with medication adherence in older asthmatics. J Gen Intern Med.

[10] Heluf H, Assefa N, Dessie Y, Tamiru D, Goshu AT, Fekadu G (2022). Adherence to anti-asthma medications among adult asthmatic patients in Eastern Ethiopia: A multi-center cross-sectional study. PLoS One.

[11] Mukherjee M, Stoddart A, Gupta RP (2016). The epidemiology, healthcare and societal burden and costs of asthma in the UK and its member nations: Analyses of standalone and linked national databases. BMC Med.

[12] Mäkelä MJ, Backer V, Hedegaard M, Larsson K (2013). Adherence to inhaled therapies, health outcomes and costs in patients with asthma and COPD. Respir Med.

[13] Kaplan A, Price D (2020). Treatment adherence in adolescents with asthma. J Asthma Allergy.

[14] Jansen EM, van de Hei SJ (2021). Global burden of medication non-adherence in chronic obstructive pulmonary disease (COPD) and asthma: A narrative review of the clinical and economic case for smart inhalers. J Thorac Dis.

[15] Ma J, Sun X, Wang X, Liu B, Shi K (2023). Factors Affecting Patient Adherence to Inhalation Therapy: An Application of SEIPS Model 2.0. Patient Prefer Adherence.

[16] Busse WW, Kraft M (2022). Current unmet needs and potential solutions to uncontrolled asthma. Eur Respir Rev.

[17] Belachew EA, Netere AK, Sendekie AK (2022). Medication regimen complexity and its impact on medication adherence and asthma control among patients with asthma in Ethiopian referral hospitals. Asthma Res Pract.

[18] Ahmad A, Sorensen K (2016). Enabling and hindering factors influencing adherence to asthma treatment among adolescents: A systematic literature review. J Asthma [Internet].

[19] Horne R, Chapman SCE, Parham R, Freemantle N, Forbes A, Cooper V (2013). Understanding patients' adherence-related Beliefs about Medicines prescribed for long-term conditions: A meta-analytic review of the Necessity-Concerns Framework. PLoS One.

[20] Zhang X, Ding R, Zhang Z, Chen M, Yin Y, Quint JK (2023). Medication Adherence in People with Asthma: A Qualitative Systematic Review of Patient and Health Professional Perspectives. J Asthma Allergy.

[21] Chan AHY, Katzer CB, Pike J, Small M, Horne R (2022). Medication beliefs, adherence, and outcomes in people with asthma: The importance of treatment beliefs in understanding inhaled corticosteroid nonadherence—a retrospective analysis of a real-world data set. J Allergy Clin Immunol Glob.

[22] Panahi S, Rathi N, Hurley J, Sundrud J, Lucero M, Kamimura A (2022). Patient Adherence to Health Care Provider Recommendations and Medication among Free Clinic Patients. J Patient Exp.

[23] Horne R, Weinman J, Hankins M (1999). The beliefs about medicines questionnaire: The development and evaluation of a new method for assessing the cognitive representation of medication. Psychol Heal.

[24] Cohen JL, Mann DM, Wisnivesky JP (2009). Assessing the validity of self-reported medication adherence among inner-city asthmatic adults: the Medication Adherence Report Scale for Asthma. Ann Allergy, Asthma Immunol.

[25] Nassar RI, Saini B, Obeidat NM, Basheti IA (2022). Development and validation of the Adherence to Asthma Medication Questionnaire (AAMQ). Pharm Pract (Granada).

[26] Dima AL, van Ganse E, Laforest L, Texier N, de Bruin M (2017). Measuring medication adherence in asthma: Development of a novel self-report tool. Psychol Heal.

[27] Plaza V, Fernández-Rodríguez C, Melero C (2016). Validation of the “Test of the Adherence to Inhalers” (TAI) for Asthma and COPD Patients. J Aerosol Med Pulm Drug Deliv.

[28] Alsous M, Alhalaiqa F, Farha RA, Jalil MA, Mcelnay J, Horne R (2017). Reliability & validity of Arabic translation of Medication Adherence Report Scale (MARS) & Beliefs about Medication Questionnaire (BMQ)±specific for use in children & their Parents. PLoS One.

[29] Tangirala NC, O'Conor R, Wolf MS, Wisnivesky JP, Federman AD (2020). Validity of the Medication Adherence Rating Scale for Adherence to Inhaled Corticosteroids among Older Adults with Asthma or Chronic Obstructive Pulmonary Disease. COPD J Chronic Obstr Pulm Dis.

[30] Helms JE, Henze KT, Sass TL, Mifsud VA (2006). Treating Cronbach's Alpha Reliability Coefficients as Data in Counseling Research. Couns Psychol.

[31] Al-Qerem W, Al Bawab AQ, Abusara O, Alkhatib N, Horne R (2022). Validation of the Arabic version of medication adherence report scale questionnaire and beliefs about medication - specific questionnaire: A factor analysis study. PLoS One.

[32] Arıkan H, Duman D, Kargin F (2018). Cross-cultural adaptation and validation of beliefs about medicines questionnaire on asthma and chronic obstructive pulmonary disease patients. Turkish Thorac J.

[33] Liu C, Tham CW, De Roza J, Chong BY, Koh YL, Tan NC (2020). The association between beliefs and adherence to inhaled controller medication among older adults with asthma: A cross-sectional study in primary care. Patient Prefer Adherence.

[34] Foot H, La Caze A, Gujral G, Cottrell N (2016). The necessity-concerns framework predicts adherence to medication in multiple illness conditions: A meta-analysis. Patient Educ Couns.

[35] Chan AHY, Horne R, Hankins M, Chisari C (2020). The Medication Adherence Report Scale: A measurement tool for eliciting patients' reports of nonadherence. Br J Clin Pharmacol.

[36] Foot H, La Caze A, Cottrell N (2017). Identifying the relationship between beliefs and medication adherence in asthma. Ann Allergy Asthma Immunol.

